# The spread of presaccadic attention depends on the spatial configuration of the visual scene

**DOI:** 10.1038/s41598-019-50541-1

**Published:** 2019-10-01

**Authors:** Martin Szinte, Michael Puntiroli, Heiner Deubel

**Affiliations:** 1 Institut de Neurosciences de la Timone, Centre National de la Recherche Scientifique, UMR 7289, Marseille, 13005 France; 2 Spinoza Centre for Neuroimaging, Royal Dutch Academy of Sciences, Amsterdam, Netherlands; 3 Institute of Management, Université de Neuchâtel, Neuchâtel, Switzerland; 4 Allgemeine und Experimentelle Psychologie, Ludwig-Maximilians-Universität München, Munich, Germany

**Keywords:** Attention, Sensory processing, Object vision, Human behaviour

## Abstract

When preparing a saccade, attentional resources are focused at the saccade target and its immediate vicinity. Here we show that this does not hold true when saccades are prepared toward a recently extinguished target. We obtained detailed maps of orientation sensitivity when participants prepared a saccade toward a target that either remained on the screen or disappeared before the eyes moved. We found that attention was mainly focused on the immediate surround of the visible target and spread to more peripheral locations as a function of the distance from the cue and the delay between the target’s disappearance and the saccade. Interestingly, this spread was not accompanied with a spread of the saccade endpoint. These results suggest that presaccadic attention and saccade programming are two distinct processes that can be dissociated as a function of their interaction with the spatial configuration of the visual scene.

## Introduction

To efficiently make sense of our rich visual environment, the visual system gained the ability to selectively process the most salient information^1^. This selection is, however, limited by the architecture of the visual system itself^2^. To compensate for the low visual resolution in peripheral vision, selection can either be achieved by shifting high resolution central vision to peripheral objects of interest by means of saccades (overt attention), or by shifting spatial attention while keeping the eyes steady (covert attention). Indeed, both cases result in the deployment of attention resources, leading to spatially localized gains in reaction time e.g.^3,4^, in visual sensitivity e.g.^5,6^ and in neural activity e.g.^7,8^.

Interestingly, saccades are preceded by a mandatory shift of attention toward the saccade target e.g.^6,9^. This shift is frequently used as primary evidence in favor of the premotor theory of attention^10,11^, a theory in which it is assumed that both overt and covert attention rely on the pre-activation of the oculomotor system.

Indeed, the presaccadic shift of attention is bound to the onset of the saccade with strong influence on visual sensitivity observed in the last 150 ms preceding its onset^12–15^. Taking advantage of the natural tendency of saccade to undershoot their target, Deubel & Schneider^6^ showed that attention was directed to the intended saccade goal rather than toward the saccade endpoint. Comparably, averaging saccades, eye movements involuntarily executed in between two targets, were not associated with a presaccadic shift of attention toward their endpoints^16^. Instead, they reflect a shared deployment of attention toward the two potential saccade targets^16^. These effects thus argue against the premotor theory of attention, as the planned saccades should have been spatially associated with the effective endpoint of the saccade.

Importantly, all the effects mentioned above rely on the high spatial specificity of the presaccadic shift of attention. For example, presaccadic attention was shown to be restricted to the saccade target and did not spread to neighboring placeholders located 1 degree of visual angle (dva) apart, that is at a distance of 20% of the executed saccade^6^. Similar effects were replicated in studies using different arrangements of visual placeholders positioned at comparable distances e.g.^16–20^. But, do these effects reflect the precision of the spatial deployment of the presaccadic shift of attention or the use of a structured visual scene for their assessment? Puntiroli and colleagues^21^ recently tested the influence of a structured visual field composed of several placeholders on the deployment of attention. They observed that the presaccadic shift of attention reduces potential masking effects that flankers can have on the saccade target. But the presaccadic shift of attention was found in both dense and sparse displays, with a strong effect also observed when the scene was composed solely of a saccade target e.g.^13,22,23^. Although the presaccadic shift of attention may reduce the influence of the object toward which the eyes are not directed to (e.g., flankers), it remains entirely unclear whether the spatial precision of this effect depends on the presence of the visual placeholder that constitutes the saccade target. Is presaccadic attention a mechanism driven by the saccade plan or by the presence of the target at the saccade landing point?

To answer this question, we will first have to clarify how the spatial spread of attention can be assessed behaviorally. Different authors have measured the spatial spread of attentional benefits through the evaluation of reaction time or sensitivity change at multiple locations surrounding a cue. When testing covert attention during fixation, attentional field sizes vary in function of the task difficulty^24^. By increasing the difficulty of the tasks by masking the targets^6,18,25,26^, or by controlling for visual eccentricity effects^27^, it was shown that the attentional spread was narrowly concentrated, with benefits limited to a few degrees of visual angle surrounding a covertly attended cue or a saccade target.

Using a novel visual sensitivity mapping paradigm, we evaluated the spatial extent of attentional benefits before a saccade made toward a cue. As expected, we found that highest sensitivity was concentrated at the cue and its immediate surrounds when it remained visible. To evaluate the influence of the cue itself, we made it disappear at different times before the saccade. Under such conditions, sensitivity benefits spread to more peripheral locations. This result suggests that the deployment of presaccadic attention interacts with the spatial configuration of the saccade target itself. Importantly, with our dual task paradigm we could evaluate whether this spread of attention was accompanied with a spread of saccade landing points. Contrary to what would have been expected if presaccadic attention relied on the pre-activation of a motor plan, we found that the observed spread of attention after the cue disappearance could not be explained by a spread of saccade endpoints.

## Results

**Figure 1:**
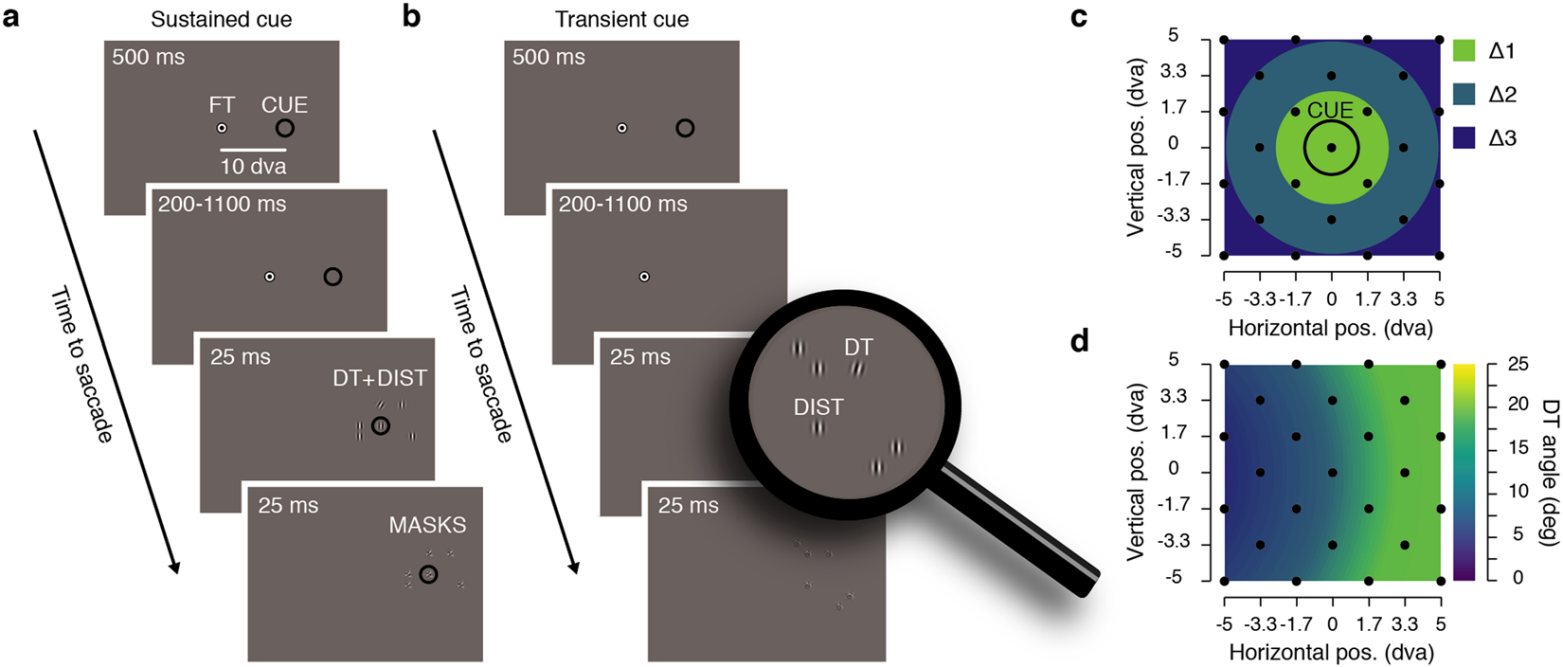
Experimental procedure. (**a**) Sustained cue condition. Participants prepared a saccade from the fixation target (FT) to a visual cue (CUE) presented continuously on the screen throughout the trial. Participants were instructed to saccade toward the center of the cue at the offset of the FT, which occurred between 700 and 1600 ms after the cue onset. Just before the saccade, a discrimination target was shown (DT, 25 ms clockwise or counterclockwise tilted Gabor) together with 5 distractors (DIST, vertical Gabors) and followed by 6 overlaying masks (MASKS, 25 ms noise patches). (**b**) Transient cue condition. Participants prepared a saccade from the FT to a CUE presented transiently (500 ms). Participants saccade at the offset of the FT which occurred between 200 and 1100 ms after the cue offset. (**c**) On each trial the position of the DT and of the distractors were randomly picked between 25 possible positions (black dots), homogeneously covering a 10° by 10° map centered on the CUE. (**d**) Before the main saccade task, we determined at different eccentricities from the FT, the necessary DT angle leading to a correct discrimination level of 80%. The graph shows averaged DT angle (n = 12) interpolated across the different DT eccentricities from the FT (see Method). DT angle is shown via the color scale, leftward saccade trials were mirrored relative to the FT.

Our goal was to determine the spatial distribution of attention when participants prepared a saccade toward a cue that either remained on the screen, or had recently disappeared. To this end, we probed attention by presenting a discrimination target at one of various locations surrounding the cue (Fig. 1). Through the use of a threshold task, we kept discrimination performance homogeneously high across space despite the fact that discrimination targets appeared at several eccentricities from the fixation target (see Methods and Fig. 1d). Then, to study the spatio-temporal dynamics of sensitivity following the disappearance of the cue, we systematically varied the delay between the initiation of the saccade and the disappearance of the cue.

We first verified that the presentation of the discrimination target itself did not systematically influence oculomotor behavior, suggesting then that our probe would have influenced our assessment of sensitivity. We did not find any differences with respect to saccade latency when comparing trials with and without the presentation of a discrimination target (4% of trials were without discrimination target, present: 201.77 ± 2.84 ms vs. absent: 199.96 ± 7.12 ms, *p* = 0.1726) and only a slight change in saccade amplitude (present: 9.67 ± 0.17° vs. absent: 9.77 ± 0.40°, *p* < 0.0366), therefore validating our procedure.

**Figure 2:**
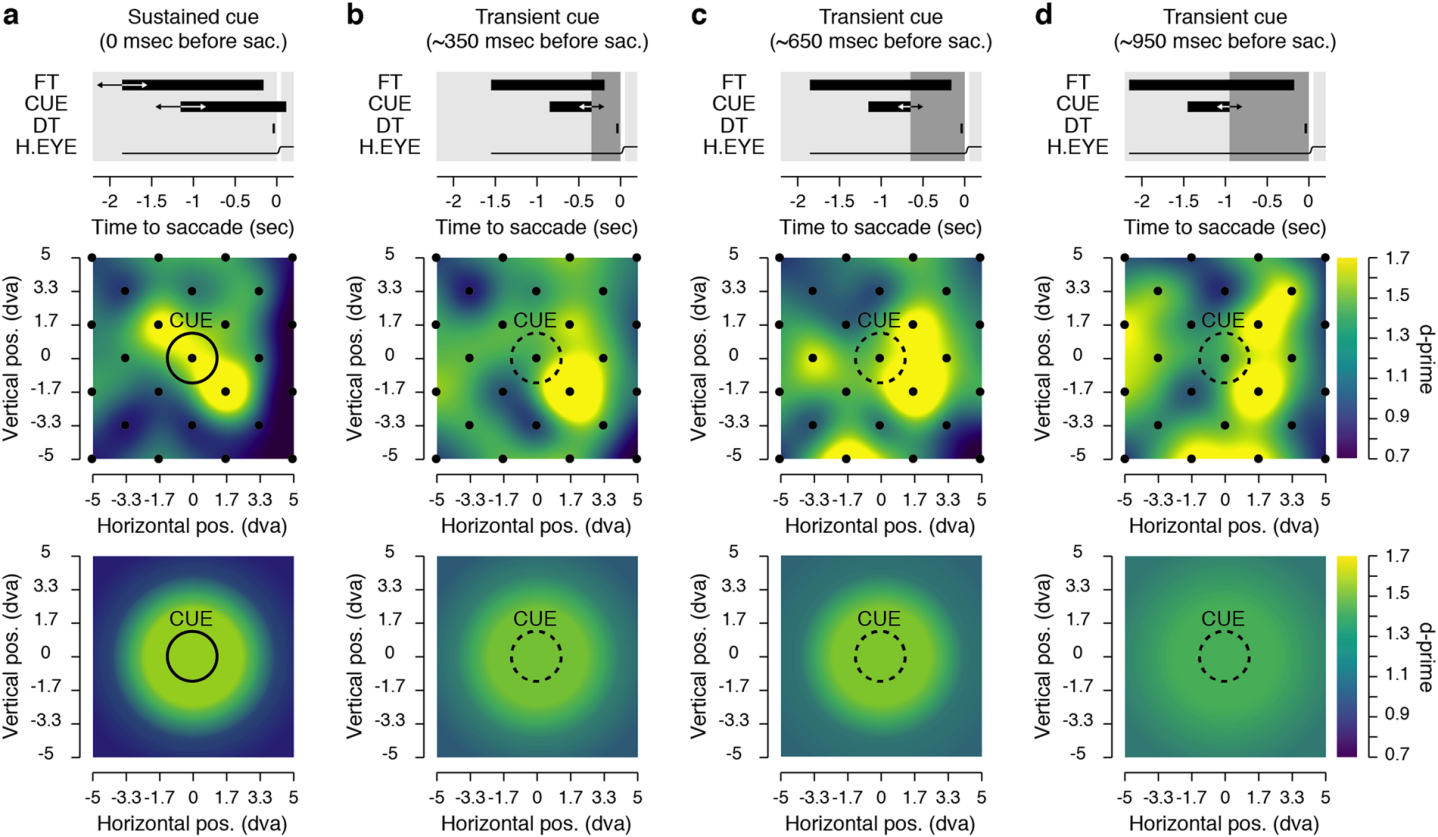
Presaccadic sensitivity maps. Each graph shows average sensitivity across all participants (see Materials and Methods) gathered either at 25 positions individually (middle row) or grouped in 3 distances (Δ1, Δ2 and Δ3, bottom row) surrounding the saccade cue (CUE). The top row of each panel describes the time course relative to the saccade onset of the fixation target (FT), the cue (CUE), the discrimination target (DT) and the horizontal eye position (H. EYE). Data are shown for the sustained cue condition (**a**) and the transient cue conditions (**b**–**d**). The transient cue condition is binned in three equal groups of trials where the cue offset preceded the saccade onset by approximately 350 (**b**), 650 (c) or 950 ms (**d**). Averaged sensitivity (d′) is shown via the color scale.

We next obtained maps of visual sensitivity, reflecting participants’ ability to correctly report the orientation of the discrimination target presented at different distances from the cue. Figure 2 shows sensitivity maps obtained across participants by presenting discrimination targets just before the saccade at 25 different positions (see Fig. 1c), for trials in which the cue remained on the screen (Fig. 2a) and trials in which it disappeared between 200 ms and 1100 ms before the saccade (Fig. 2b–d). We thus measured sensitivity across 100 dva^2^ with a substantial but still limited number of trials per participants and condition for each of the tested positions (40.76 ± 1.26 trials). The constraints of studying human volunteers (in total the experiment lasted between 5 and 6 h) made it hard to reduce the high level of noise inherent in the production of individual sensitivity maps (see Supplementary Fig. S1). Nevertheless, when sensitivity maps are averaged across participants one can appreciate the effects of the cue on the allocation of sensitivity. Indeed, these maps show that sensitivity benefits were more pronounced toward the immediate contour of the cue and subsequently spread as a function of the distance from the cue, more and more, as the delay between the saccade onset and the cue offset increased. These effects were systematically analyzed by combining the 25 tested positions into 3 groups of discrimination target distances from the cue (see Δ1, Δ2 and Δ3 in Fig. 1c). Such binning of the data, which was necessary to summarize our results and to reduce the noise level, can be considered arbitrary. However, it allows presaccadic sensitivity to be assessed at 3 different distances, corresponding to positions from the cue included within the range of 24% (Δ1), 47% (Δ2) and 71% (Δ3) of the saccade size. The first proportion corresponds to the distance used in the first paper about that issue^6^, the new distances used being then about two to three times this amount.

**Figure 3:**
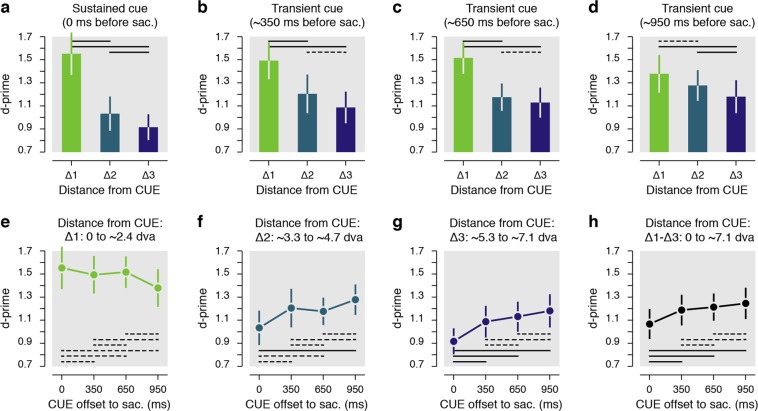
Results grouped from distance from the cue. (**a**–**d**) Presaccadic sensitivity as a function of the distance from the cue center (Δ1-Δ3). Data are shown for the sustained cue (**a**) and the transient cue conditions (**b**–**d**). The transient cue condition is binned in three equal groups of trials where the cue offset precede the saccade by approximately 350 ms (**b**), 650 ms (**c**) or 950 ms (d). (**e**–**h**) Presaccadic sensitivity as a function of the duration between the cue offset and the saccade onset. Data are shown separately for three main distances of the DT from the CUE center (**e**–**g**) or for all trials irrespective of their distance from the cue (**h**). Note that overall sensitivity results (**h**) is not directly the means of the sensitivity observed at different distances from the cue (**e**–**g**) as there was not the same number of trials played at each analyzed distance from the cue. Error bars show SEM, dashed and full lines represent nonsignificant (*p* > 0.05) and significant (*p* < 0.05) comparisons, respectively. See Supplementary Fig. S2 and Supplementary Table 1 for individual participants’ results.

Results reveal that within the trials in which the cue remained on the screen (Fig. 3a), performance was best for discrimination targets presented within ~2.4° surrounding the cue (Δ1: d′ = 1.55 ± 0.18 vs. Δ2: d′ = 1.03 ± 0.15, *p* < 0.0001; Δ1 vs. Δ3: d′ = 0.92 ± 0.11, *p* < 0.0001), with sensitivity at the immediate surround of the cue being approximately 63% higher compared to discrimination targets shown at further distances (Δ1/Δ2: 159.54 ± 14.65%, Δ1/Δ3: 170.97 ± 13.14%). These results are in line with previous evidence showing that the presaccadic performance is limited toward the closest positions surrounding a saccade target^6^.

Next, we found a similar pattern of results when, rather than remaining continuously on the screen, the cue was extinguished approximately 350 ms before the saccade (Fig. 3b–d). Within these trials we observed a similar distribution of presaccadic sensitivity with best performance limited within the immediate contour of the cue (Δ1: d′ = 1.49 ± 0.16 vs. Δ2: d′ = 1.21 ± 0.17, *p* < 0.0406; Δ1 vs. Δ3: d′ = 1.09 ± 0.14, *p* < 0.0004), and with an increase of sensitivity of approximately 42 and 46% when compared with performance at the two further distances respectively (Δ1/Δ2: 142.42 ± 22.43%, Δ1/Δ3: 146.49 ± 19.17%). These results seem to indicate that the offset of the cue had no significant influence on the allocation of sensitivity before a saccade, at least within the first 350 ms following its offset. Next, when the cue had disappeared from the screen approximately 650 ms before saccade start (Fig. 3c), we found that targets were better discriminated if they were presented within the first distance from the cue (Δ1: d′ = 1.52 ± 0.14 vs. Δ2: d′ = 1.18 ± 0.12, *p* < 0.0008; Δ1 vs. Δ3: d′ = 1.13 ± 0.13, *p* < 0.0002), with, however, a reduced spatial cueing effect (Δ1/Δ2: 137.98 ± 17.56%, Δ1/Δ3: 146.56 ± 13.83%). The trend toward a broader spread of higher sensitivity became evident when the cue had disappeared approximately 950 ms before the saccade (Fig. 3d). For these trials, the sensitivity difference between first and the second distances from the cue was no longer significant (Δ1: d′ = 1.38 ± 0.16 vs. Δ2: d′ = 1.28 ± 0.13, *p* = 0.3608) and cueing effects between the first and the two other distances were strongly reduced (Δ1/Δ2: 108.03 ± 11.52%). Also, within these trials, performance for discrimination targets presented between ~3.3° and ~4.7° from the cue (Δ2) was now slightly better than for trials in which they were shown even further away (Δ3) from the cue (Δ2 vs. Δ3: d′ = 1.18 ± 0.14, *p* < 0.0484); an effect observed for trials in which the cue remained onscreen (*p* < 0.0186) but not for trials in which the cued disappeared less than approximately 950 ms before the saccade (all *ps* > 0.4964).

The results above suggest that attentional resources were, in general, drawn toward the cue and its close proximity, at least when it remained on the screen, or when it disappeared less than 950 ms before the saccade. To capture the time course of the spread, we compared sensitivity gathered at each of discrimination target-to-cue distances (Δ1-Δ3) in function of the delay between the saccade onset and the cue offset. We considered trials in which the cue remained on the screen as the shortest delay (t0, “t” corresponding to the cue offset to the saccade onset time). We found that sensitivity for discrimination targets shown at the cue’s immediate contour (Δ1) remained at a very similar level over the tested delays (0.8324 > *ps* > 0.1102, see dashed lines in Fig. 3e). On the other hand, sensitivity for discrimination targets shown at positions more than 2.4° from the cue (Δ2 and Δ3) gradually improved as the time from cue offset increased (Fig. 3f–h). In particular, we found a significant improvement of sensitivity for discrimination targets shown between ~3.3° and ~4.7° from the cue (Δ2, Fig. 3f) when comparing trials where the cue remained on the screen to trials when the cue offset preceded the saccade by approximately 950 ms (t0: d′ = 1.03 ± 0.15 vs. t950: d′ = 1.28 ± 0.13, *p* < 0.0001), but not when the delay was shorter (i.e. t350 and t650: 0.1256 > *ps* > 0.1076). Such a spread of resources for discrimination targets shown at the greatest tested distance from the cue (Δ3, Fig. 3g) was visible even earlier in time (i.e. for shorter cue offset to the saccade). Significant differences were, in fact, found in trials where the cue disappeared approximately 350 ms (t0: d′ = 0.92 ± 0.11 vs. t350: d′ = 1.09 ± 0.14, *p* < 0.0098) and even earlier relative to the saccade onset (i.e. t650 and t950: both *ps* < 0.0001). Interestingly, when considering all discrimination target positions together (Fig. 3h), we found that sensitivity increased as a function of the delay between the saccade onset and the cue offset. Overall sensitivity (computed as the average across the 25 possible positions of the discrimination target) increased by approximately 27% when comparing trials in which the cue disappeared about a second before the saccade to those in which the cue remained on the screen (t950/t0: 126.67 ± 10.62%). Furthermore, this increase of sensitivity was already significant when comparing trials where the cue remained on the screen with trials where the cue was extinguished approximately 350 ms before the saccade (t0: d′ = 1.07 ± 0.13 vs. t350: d′ = 1.19 ± 0.13, *p* < 0.0490) or even earlier (t0 vs. t650: d′ = 1.21 ± 0.12, *p* < 0.0022, t0 vs. t950: d′ = 1.25 ± 0.14, *p* < 0.0001). Note that in the condition where the cue remained on the screen, participants were still instructed to initiate their saccade at the offset of the fixation target which occurred across the same range of duration than in the transient cue condition. We, however, considered these trials as a single condition as we were here interested by the temporal dynamics following the cue offset. Nevertheless, we verified that sensitivity was not affected by this waiting period preceding the saccade. To do so, we analyzed these trials as a function of the cue onset relative to the saccade. We found the same pattern of sensitivity irrespective of the waiting period before the saccade, a pattern almost identical to what observed when these trials were considered as a single condition. In detail, we found highest sensitivity for discrimination targets presented within ~2.4° surrounding the cue both when the cue onset occurred between 700 and 1150 ms (Δ1: d′ = 1.57 ± 0.17 vs. Δ2: d′ = 0.96 ± 0.17, *p* < 0.0001; Δ1 vs. Δ3: d′ = 0.93 ± 0.10, *p* < 0.0001) or between 1150 and 1600 ms before the saccade (Δ1: d′ = 1.58 ± 0.22 vs. Δ2: d′ = 1.08 ± 0.16, *p* = 0.0094; Δ1 vs. Δ3: d′ = 0.93 ± 0.13, *p* < 0.0001). Moreover, irrespective of the distance of the cue (Δ1-Δ3) we didn’t find any significant difference between these two cue onset times relative to the saccade (1.0 > *ps* > 0.3880).

Altogether, we observed that presaccadic processing resources were deployed to the cue and maintained within its close proximity even one second after its disappearance. Moreover, the cue’s disappearance was quickly followed by an improvement of sensitivity at distances further away from it.

**Figure 4:**
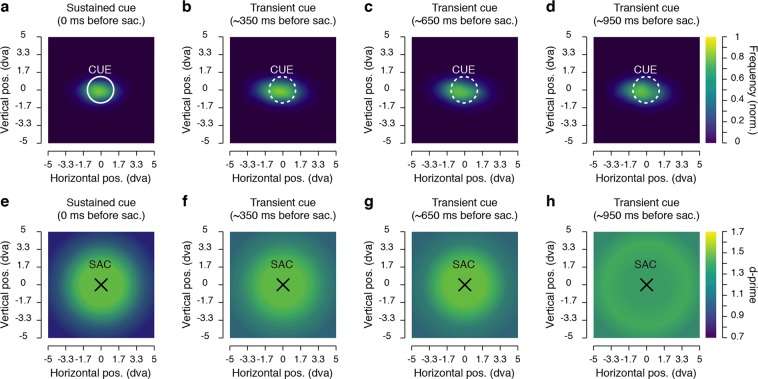
Saccade endpoint maps and sensitivity maps relative to saccade endpoint. (**a**–**d**) Normalized saccade landing maps averaged across participants. Data are shown for the sustained cue (**a**) and the transient cue conditions where the cue offset preceded the saccade by approximately 350 ms (**b**), 650 ms (**c**) or 950 ms (**d**). (**e**–**h**) Each graph shows average sensitivity grouped in 3 distances (β1, β2 and β3) surrounding each saccade endpoint (SAC). Data are shown for the sustained cue (**e**) and the transient cue conditions, when the cue offset preceded the saccade by approximately 350 ms (**f**), 650 ms (**g**) or 950 ms (**h**). Conventions are as in Fig. 2.

Can these results be explained by the fact that, after a rather long delay after the cue disappearance, participants lost track of its location? Because we investigated changes of sensitivity in a saccade task, our design allowed us to use saccade metrics to test this alternative explanation. First, we looked at whether the spread of resources was accompanied by a comparable spread of saccade endpoints. Figure 4a–d shows the normalized saccade landing frequency for the different delays between the cue offset relative and the saccade onset. From these graphs, one can appreciate the absence of any strong difference in the frequency of saccade landing, which would be expected from the spread of sensitivity observed above. The disappearance of the cue, nevertheless, had a clear influence on the saccades. In particular, we found that saccades were more accurate (accuracy assessed as the absolute distance between the saccade offset and the saccade target, t0: 1.24 ± 0.04° vs. t350-950: 1.47 ± 0.05°, *p* < 0.0001) and more precise (precision assessed as the standard deviation of the accuracy, t0: 0.68 ± 0.02° vs. t350-950: 0.80 ± 0.02°, *p* < 0.0001) when the cue remained on the screen compared to trials where it disappeared from the screen. We attributed the increased spread of saccade endpoints to the offset of the cue itself^28^, and the lack of visual feedback just before saccade onset. In fact, contrary to what we found for sensitivity, when the cue disappeared from the screen both saccade accuracy and precision didn’t change as a function of the delay between cue offset and saccade onset (0.5882 > *ps* > 0.1144).

**Figure 5:**
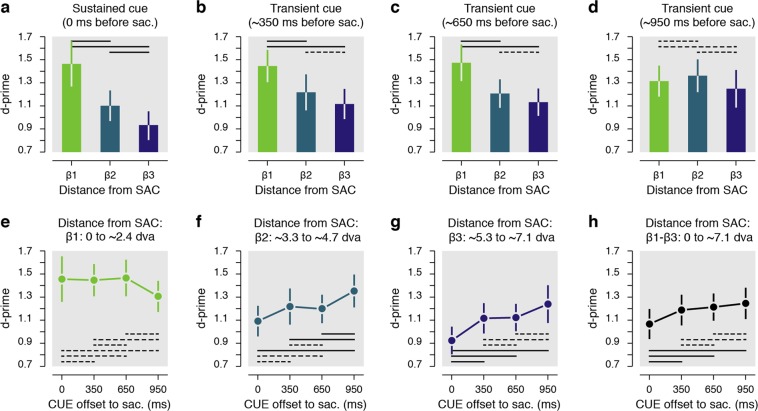
Results grouped based on the attentional probe’s distance from the saccade endpoint. (**a**–**d**) Presaccadic sensitivity as a function of the distance from the saccade endpoint (β1–β3). Data are shown for the sustained cue (**a**) and the transient cue conditions (**b**–**d**). The transient cue condition is binned in three equal groups of trials where the cue offset precedes the saccade by approximately 350 ms (**b**), 650 ms (**c**) or 950 ms (d). (**e**–**h**) Presaccadic sensitivity as a function of the duration between the cue offset and the saccade onset. Data are shown separately for three main distances of the DT from the saccade endpoint (**e**–**g**) and for all trials, irrespective of the distance between the DT and the saccade endpoint (**h**). Error bars show SEM, dashed and full lines represent nonsignificant (*p* > 0.05) and significant (*p* < 0.05) comparisons, respectively. See Supplementary Fig. S3 and Supplementary Table 2 for individual participant results.

Next, using saccade endpoint coordinates, we re-encoded the discrimination target positions in order to obtain sensitivity maps relative to the saccade endpoints, rather than relative to the cue position (Fig. 4e–h). We hypothesized that if the spread of sensitivity was due to a spread in saccade landing, then, by correcting the discrimination target coordinates for the saccade endpoints, the differences in sensitivity observed for different delays between cue offset and saccade onset should no longer be found, or at least be reduced. This was not the case. Instead, we found a very similar spread of sensitivity as above, with sustained effect over time observed at the immediate contour of the saccade endpoints (Fig. 5a, β1: 0.9776 > *ps* > 0.1782), and a similar spread of sensitivity across temporal delays for the other distances from the saccade endpoint (Fig. 5b–d). Also, as for the main analysis relative to the cue location, we found a significant improvement of sensitivity for discrimination targets shown between ~3.3° and ~4.7° from the saccade endpoint (β2, Fig. 5f) when comparing trials where the cue remained on the screen to trials where the cue offset preceded the saccade by approximately 950 ms (t0: d′ = 1.09 ± 0.13 vs. t950: d′ = 1.35 ± 0.14, *p* < 0.0044), but not if this delay was shorter (i.e. t350 and t650: 0.9776 > *ps* > 0.9410). Moreover, we found an earlier spread of sensitivity for discrimination targets shown at the greatest tested distances from the saccade endpoint (β3, Fig. 5g), with significant differences found for trials in which the cue disappeared approximately 350 ms (t0: d′ = 0.92 ± 0.12 vs. t350: d′ = 1.12 ± 0.13, *p* < 0.0170) and after longer delays (i.e. t650 and t950: both 0.0080 > *ps* > 0.0001). This illustrates a clear dissociation of saccade preparation and sensitivity, with the spread of saccadic endpoints not mirroring the spread of sensitivity.

## Discussion

We observed that presaccadic attention remained bound to the cue location, and its immediate surrounds, before a saccade to a continuously present cue. Once the cue disappeared, we observed a spread of attention to more peripheral locations further away from the memorized target. This spread followed specific temporal dynamics. Notably, we found a modest improvement in sensitivity at the farthest tested distance from the cue starting early after its offset. At a closer distance from the cue, we observed a strong improvement in sensitivity beginning significantly later. Interestingly, the spread of attention was never accompanied by a significant reduction in sensitivity at the most central tested locations, close to the cue location. Here performance was always highest, irrespective of whether the cue remained visible or disappeared. In other words, we did not observe a trade-off of attentional resources, where a spread of attention would lead to a dilution of attention resources. Instead, we found that sensitivity increased overall as the delay between the saccade and the cue offset grew. Furthermore, the spread of attention could not be explained by a spread of saccades toward the target location. Although saccade accuracy slightly decreased when saccades were executed toward a cue that was no longer present, the sensitivity maps were unchanged after we corrected the tested position by the trial-by-trial retinal error, suggesting that actual saccade landing had no influence on the spread of attention. Our findings suggest that the deployment of attention before saccade depends on the presence of the cue, with a concentration of attentional resources confined within ~2.4° when the cue remained on the screen and an undifferentiated spread of attention within ~4.7° around a cue that has disappeared about a second ago.

The extent to which an exogenous cue modulates the deployment of spatial attention has been investigated previously, with outcomes mostly reflecting the difficulty of the task^24^. Our new paradigm allowed us to adjust the task difficulty for each participant and to control for visual eccentricity effects^29^, two essential requirements for assessing visual sensitivity over space^27^. By combining a discrimination and a saccade task, we were able to verify, through saccade metrics, that our measure of attention was not affected over space by the use of transient stimuli^6^. By analyzing the distribution of saccade endpoints, we demonstrated that participants effectively kept track of the cued position at all the tested delays.

We observed that the modulation of attention was tightly limited to the intended saccade cue and its immediate surrounds when the target remained on the screen, with highest sensitivity observed at roughly an equivalent distance to that observed in previous reports^6^. While our findings match those of studies which investigated the allocation of attention with a continuously presented cue^6,13,14,18,20,22,23,30,31^, these studies were all using a structured visual field of visual placeholders. Puntiroli and colleagues^21^ is to our knowledge the only study in which the effect of visual placeholders on the presaccadic shift of attention was investigated. They measured the relative performance in discriminating a cross displayed at three possible placeholders, the saccade target and two distractors, either present or absent of the screen in different blocks of trials. They found that the masking effect of the flankers was reduced by the presaccadic shift of attention. Contrary to their study, we here did not try to avoid the use of a placeholder, as the screen can act itself as a placeholder. Moreover, we used a threshold procedure at fixation, in which we presented the same cue at all the locations tested. Under such conditions, any masking of the cue itself should have been canceled. Despite using visual stimuli to measure attention we ensured these were presented only immediately before the saccades, at a time when the eye movements could no longer be stopped^32,33^ and when attention had already been allocated at the saccade target^14^. We therefore captured for the first time the allocation of presaccadic attention in an unstructured visual field and observed its spread from the cue center to its periphery. We found that, contrary to the narrow allocation of presaccadic attention for a structured visual field, attention dynamically spread over space as a function of the delay between cue-offset and saccade onset. These effects are in contrast with a recent report in which the disappearance of a briefly presented cue, during a fixation task, lead to slower reaction times in detecting the presence of a flashed target on a black screen in the absence of placeholders^34^. Contrary to these effects, we observed an overall increase in sensitivity after the disappearance of the target. This difference could reflect the difficulty in drawing conclusions about the deployment of attention based on the use of reaction times to supra-threshold stimuli^26^. Further studies should evaluate the influence of visual placeholders when measuring the deployment of attention to determine the influence the structure of the visual field on both overt and covert attention mechanisms.

Sensitivity was better than chance at more peripheral positions from the cue, suggesting that presaccadic attention was not exclusively allocated to the saccade target^18,30,31^. We attribute his effect to the use of transient discrimination targets. Indeed, to evaluate the deployment of attention in an unstructured visual field we used transient discrimination targets, that to some extent capture by themselves attention^35,36^. With such a protocol, performance was maintained high across space, allowing us to make interpretations only on the modulation of presaccadic attention across conditions, rather than to draw conclusions about the absolute deployment of attention.

We studied the deployment of attention before the execution of saccades across different conditions. Thus, we measured behaviorally the consequences of feedback activations of the priority maps (Frontal Eye Fields —FEF—, Superior Colliculus —SC— and the parietal cortex –LIP–) onto the feature maps (V1-V4) of the visual cortex^37,38^. Different studies indeed have demonstrated strong connectivity between oculomotor and feature maps preceding the execution of a saccade^39–41^. These studies demonstrated the existence of a strong link between oculomotor and attentional processes. Nevertheless, they did not necessarily support the existence of an activity of the oculomotor system preceding any deployment of attention as suggested by the premotor theory of attention^10,11^. Here, following the offset of visual inputs, we observed a large spread of presaccadic attention that was not accompanied by a spread of the saccade endpoints. We hypothesize that these effects are the consequence of a neural dissociation between visual and motor cells within the priority maps^42,43^. Indeed, it was shown that motor cells within FEF or SC stayed completely silent during an attention task made at fixation^39,44,45^, while visual and visuo-motor cells displayed sustained attentional effects. Moreover, it has been shown that LIP inactivation can disrupt attentional capabilities while leaving oculomotor processes intact^46,47^, contrary to inactivation of FEF and SC^48,49^, which tend to also disrupt eye movements. If such neural dissociation would explain our findings, the spread of presaccadic attention observed without the presence of the cue could reflect the necessity of constant inputs for the proper precise functioning of visual neurons, on the contrary of visuo-motor and motor cells which could maintain a saccade plan over long duration^50^. While electrophysiological studies are needed to evaluate the mechanisms sustaining the observed spread of attention without a spread of saccades endpoints, our results speak against the main assumption of the premotor theory of attention^10,11^, as the theory does not predict a spread of attention of the kind observed here, with the saccade execution remaining unaffected.

Finally, we observed that the cue’s disappearance results in an increase of the attentional field size. These effects match with those reported in a fMRI study on delayed endogenous attention during fixation, in which Herrmann and colleagues^51^ found that the size of attention fields increases when comparing trials with placeholders to those without. As changes in attention field size constitute the core of the influential normalization model of attention^52^, future studies should make use of such a fruitful manipulation to further test its predications. As an alternative to these studies one could observe the changes in attention field size by manipulating the size of the saccade landing spread through the use of low-contrast cues in humans or by inactivating attentional centers in animal study.

Using a novel sensitivity mapping paradigm, we evaluated the spatial and temporal dynamics of presaccadic attention following the disappearance of a cue. Our results provided evidence for a spread of attentional resources occurring without a spread of saccade endpoints. These results suggest that the spatial distribution of presaccadic attention is strongly modulated by the presence of placeholders. Moreover, attention and saccade programming can be seen, in which presaccadic attention spread is to a various degree dependent on the spatial configuration of the visual scene, while the distribution of saccade endpoints is unaffected by the visual layout.

## Materials and Methods

### Ethics statement

This experiment was approved by the Ethics Committee of the Faculty for Psychology and Pedagogics of the Ludwig-Maximilians-Universität München (approval number 13_b_2015) and conducted in accordance with the Declaration of Helsinki. All participants gave written informed consent.

### Participants

Twelve students of the LMU München participated in the experiment (ages 19–29, 5 females, 1 author), for a compensation of 10 Euros per hour of testing. All participants except one author (MP) were naive as to the purpose of the study and all had normal or corrected-to-normal vision.

### Setup

Participants sat in a quiet and dimly illuminated room, with their head positioned on a chin and forehead rest. The experiment was controlled by an Apple Mac mini computer (Cupertino, CA, USA). Manual responses were recorded via a standard keyboard. The dominant eye’s gaze position was recorded and available online using an EyeLink 1000 Tower Mount (SR Research, Osgoode, ON, Canada) at a sampling rate of 1 kHz. The experimental software controlling the display, the response collection as well as the eye tracking was implemented in Matlab (The MathWorks, Natick, MA, USA), using the Psychophysics^53,54^ and EyeLink toolboxes^55^. Stimuli were presented at a viewing distance of 60 cm, on a 21-in gamma-linearized LaCie Electron 21/108 CRT screen (Paris, France) with a spatial resolution of 1024 × 768 pixels and a vertical refresh rate of 120 Hz.

### Procedure

The study was composed of a main saccade task tested in 3 to 4 experimental sessions (on different days) of about 90 minutes each (including breaks). The main task was always preceded by a threshold task at the beginning of each experimental session. Each session was composed of 2 blocks of the threshold task followed by 3 to 4 blocks of the main saccade task. All participants ran a total of 20 blocks of the main saccade task.

### Main saccade task

Each trial began with participants fixating a central fixation target forming a black (~0 cd/m^2^) and white (88 cd/m^2^) “bull’s eye” (0.4° radius) on a gray background (44 cd/m^2^). When the participant’s gaze was detected within a 3.0° radius of a virtual circle centered on the fixation target, for at least 200 ms, the trial began with a fixation period of 500 ms. After this period, a saccade cue consisting of a black (~0 cd/m^2^) outlined circle (1.25° radius, 0.1° width) was presented 10° to the right or to the left of the fixation target (see Fig. 1). The cue either stayed on the screen for a duration of 500 ms (3/4 of the trials) or remained continuously on the screen until the end of the trial (1/4 of the trials). Participants were instructed to move their eyes as quickly and as accurately as possible toward the center of the cue at the offset of the fixation target, which occurred at different times after the cue onset (see below). Following the fixation target offset, one discrimination target and five distractors were shown for a duration of 25 ms. The positions of target and distractors were randomly selected among 25 possible positions homogeneously covering a 10° by 10° map centered on the saccade cue (positions located at every second intersection of a 7 columns by 7 rows grid, see Fig. 1c). All targets were Gabor patches (frequency: 1.75 cycles per degree; 100% contrast; random phase across trials; Gaussian envelope: 0.6°). While the distractors were vertical Gabors, the discrimination target was a tilted Gabor (clockwise or counterclockwise relative to the vertical) with an angle adjusted in the threshold task for the different distances from the fixation target (see threshold task). All targets were later replaced by Gaussian pixel noise masks for a duration of 25 ms (made of ~0.11°-width pixels with the same Gaussian envelope as the Gabors). We didn’t present any target or mask in 4% of all the trials, in order to evaluate the influence of our stimuli on the saccade execution (note that all other analyses are based on the discrimination target present trials). At the end of each trial, participants reported the orientation of the discrimination target using the keyboard (right or left arrow keys) followed by a negative-feedback sound in the case of an incorrect response. On trials where no target was shown, participants randomly pressed one of the two response buttons, followed by a random feedback sound.

Each participant completed between 2914 and 3773 trials of the main saccade task. Correct fixation resulted from gaze being maintained within a 3.0° radius virtual circle centered on the fixation target. Correct saccades resulted from saccades landing within a 4.0° radius virtual circle centered on the cue. Note that these criteria were chosen to be lax, to avoid the possibility to filter out any spread of the saccade endpoint. Both criteria were checked online. Trials with fixation breaks or incorrect saccades were repeated at the end of each block, together with trials during which a saccade was initiated (crossing the virtual circle around the fixation target) within the first 50 ms or ended (crossing the virtual circle around the cue) after more than 350 ms following the fixation target offset (participants repeated between 114 to 973 trials across all sessions).

### Stimuli timing

The saccade signal delay (fixation target offset relative to the cue onset) was selected in order to have eye movement onset randomly interspersed between 700 and 1600 ms after the cue onset. In order to obtain this temporal range we had to account for systematic changes in saccade latency, in function of the saccade signal delay. Indeed, in a pilot experiment we observed that participants quickly learned the range of possible delays, with shorter saccade latency observed for longer signal delays and conversely (Supplementary Fig. S4). At the end of each block, we determined the slope (−0.09 ± 0.01 ms) and intercept (215.84 ± 4.88 ms) of a linear regression best describing this relationship (for the first block, we used fixed slope and intercept values of −0.5 ms and 200 ms, respectively). The saccade signal was then determined on each trial by subtracting the saccade latency estimated for each trial delay from a randomly selected duration (between 700 and 1600 ms in steps of ~8.3 ms, a screen frame). This gave us signal delays ranging between 428.92 ± 6.46 ms and 1435.33 ± 2.46 ms after the cue onset (from the trials after data pre-processing, see below).

Next, in order to probe attention in the last 100 ms preceding saccades we played the discrimination and distractors randomly between 50 and 100 ms (in steps of 25 ms), before the saccade latency, estimated for a given saccade signal delay trial. This resulted (from the trials after data pre-processing) in discrimination target offset time relative to the saccade onset of **−**48.02 ± 1.16 ms (Supplementary Fig. S4).

### Threshold task

The threshold task preceded the saccade main task at the beginning of each experimental session. This task made it possible to counteract possible learning effects and to adjust the baseline performance, for the presentation of a discrimination target at different distances from the fixation, across participants. This latter point was particularly important as it reduced the impact of eccentricity effects^29^ onto the mapping of attention benefits. Contrary to the main task, participants were instructed to keep fixation on the fixation target, which remained on the screen. Also, compared to the main task, the cue could be presented at any of the 25 positions where the discrimination targets were shown, and it remained on the screen until the end of each trial. The discrimination target always followed the cue onset by 200 ms and was always presented at the cued location. With the exception of these differences the threshold task otherwise matched the main task.

The 25 possible positions of the discrimination target and cue were subdivided into 4 equiprobable groups of distances from the fixation target (distance 1: from ~5.3° to ~7.5°; distance 2: from ~8.5**°** to ~10.5°, distance 3: from ~11.8° to ~13.7°; distance 4: from ~15.1° to ~15.8°). Following a procedure of constant stimuli, the orientation of the discrimination target varied randomly across trials between five linearly spaced steps (between ±1° and ±17° for distances 1–2; and between ±1° and ±29° for distances 3–4). Participants were instructed that the cue would always indicate the discrimination target’s location in all the trials and were told to report its orientation (clockwise or counterclockwise) at the end of each trial. They completed 2 blocks of 160 trials, and correct fixation within a 3.0° radius virtual circle centered on the fixation target was checked online. Trials with fixation breaks were immediately discarded and repeated at the end of each block.

For the four main distances from the fixation target, we individually determined, for each participant and on each experimental session, four threshold values, corresponding to the discrimination target’s angles that would lead to correct discrimination on 85% of trials. To do so, we fitted four cumulative Gaussian functions to performance gathered in the threshold blocks. These threshold angles were used in the main task for discrimination targets, played at their respective distances from the fixation target.

### Data pre-processing

Before proceeding to the analysis of the behavioral results of the main task, we scanned the recorded eye-position data offline. Saccades were detected based on their velocity distribution^56^ using a moving average over twenty subsequent eye position samples. Saccade onset and offset were detected when the velocity exceeded and fell behind the median of the moving average by 3 SDs for at least 20 ms. We included trials where a correct fixation was maintained within a 3.0° radius centered on the fixation target, where a correct saccade started at the fixation target and landed within a 4.0° radius centered on the cue and where no blink occurred during the trial. Finally, only trials in which the discrimination target offset occurred in the last 150 ms preceding saccades were included in the analysis. In total, we included 27414 trials (81.25% of the online selected trials, 69.48% of all trials played) of the main saccade task.

### Behavioral data analysis

We designed our experiments in order to have a similar number of trials between 4 main delays, defined by the offset time of the cue relative to the onset of the saccade (t). We considered the condition in which the cue remained continuously on the screen as our first delay (t0) and next determined three bins of trials; one when the cue offset occurred between 200 and 500 ms (t350), one between 500 and 800 ms (t650) and one between 800 and 1100 ms (t950) before the saccade onset. For each participant and each of these conditions, we determined the sensitivity in discriminating the orientation of the discrimination target (d′): *d*′ = *z*(hit rate) - *z*(false alarm rate). To do so, we defined a clockwise response to a clockwise discrimination target (arbitrarily) as a hit and a clockwise response to a counterclockwise discrimination target as a false alarm. Corrected performance of 99% and 1% were substituted if the observed proportion correct was equal to 100% or 0%, respectively. Performance values below the chance level (50% or d′ = 0) were transformed to negative d′ values. Sensitivity was computed either separately for each of the 25 different positions of the discrimination targets or for target positions grouped in three main distances (Δ) from the cue location (Δ1: from 0° to ~2.4°; Δ2: from ~3.3° to ~4.7°, Δ3: from ~5.3° to ~7.1°). These distances were arbitrarily defined in order to increase the power of our analyses. In our analyses we included per participants on average 40.76 ± 1.26 trials per discrimination target position and time condition, giving then 176.65 ± 5.47 trials per time condition when individual positions were combined in three main distances. We also computed sensitivity for test positions relative to the saccade landing position. To do so, for each trial we recomputed the distance between the observed saccade landing and the discrimination target coordinates individually. Later, we grouped trials into the same 3 distances as above (β), but this time from the saccade landing point (β1: from 0° to ~2.4°; β2: from ~3.3° to ~4.7°, β3: from ~5.3° to ~7.1°).

Average sensitivity maps of target discrimination (see Fig. 2a–d, middle panels) were first obtained by interpolating (triangulation-based natural neighbor interpolation) the missing values located every two intersections of the 7 columns by 7 rows grid. This was achieved by using the mean sensitivity for each participant, obtained over 25 positions of the discrimination target. Then the grid was rescaled (Lanczos resampling method) so as to obtain a finer spatial grain. These maps were then produced by drawing colored squares centered on their respective coordinates and following a linear color scale going from d′ = 0.7 to d′ = 1.7. Sensitivity maps of individual participants were prepared following the same steps but with a larger color scale going from d′ = 0 to d′ = 3 (Supplementary Fig. S1). To better compare individual participants’ results despite the difference in individual baselines, we drew sensitivity maps normalized across the 25 positions tested following this formula d′_n_ = (d′_n_ − min)/(max − min), with d′_n_ the sensitivity at a given position n, and min and max, respectively the minimum and maximum sensitivity obtained across the 25 tested positions in the specific condition (Supplementary Fig. S1). Groups of discrimination target sensitivity maps (see Fig. 2a–d bottom panels and Fig. 4e–h) were obtained by interpolating (linear interpolation) the mean sensitivity obtained over in the 3 different groups of main distances between the discrimination targets and the cue positions (Δ) or between the discrimination targets and the saccade endpoint position (β). These maps were then produced by drawing colored circles centered on the cue or saccade landing point, with a radius corresponding to their respective distances from the cue or the saccade landing point and following the same color scale used for the position sensitivity maps. A similar procedure was used to draw the threshold angle map (see Fig. 1d) this time with a linear color scale going from threshold angles of 0° to 25° and colored circles centered on the fixation target. Saccade endpoint maps (Fig. 4a–d and Supplementary Fig. S1) were obtained through the use of a bivariate kernel density estimator^57^ were first normalized relative to the total number of trials within a condition and later averaged across participants.

For statistical comparisons we drew 10,000 bootstrap samples (with replacement) from the original pair of compared values. We then calculated the difference of these bootstrapped samples and derived two-tailed *p* values from the distribution of these differences.

## Supplementary information

Supplementary file

## Data Availability

All files are available from the OSF database: https://osf.io/zmhrk/.
