# Supplementary material for: The spread of presaccadic attention depends on the spatial configuration of the visual scene
Source: Sci Rep. 2019 Oct 1;9:14034. doi: 10.1038/s41598-019-50541-1 (PMC6773758; doi:10.1038/s41598-019-50541-1)
Supplement: Supplementary file 1 — Supplementary file [file 41598_2019_50541_MOESM1_ESM.pdf]

The spread of presaccadic attention depends on the spatial configuration of the visual scene

Martin Szinte[1,2]*, Michael Puntiroli[3] & Heiner Deubel[4]

1 Institut de Neurosciences de la Timone,
Centre National de la Recherche Scientifique, UMR 7289, Marseille, France
2 Spinoza Centre for Neuroimaging,
Royal Dutch Academy of Sciences, Amsterdam, Netherlands
3 Enterprise Institute,
Université de Neuchâtel, Neuchâtel, Switzerland.
4 Allgemeine und Experimentelle Psychologie,
Ludwig-Maximilians-Universität München, Munich, Germany.

* martin.szinte@gmail.com

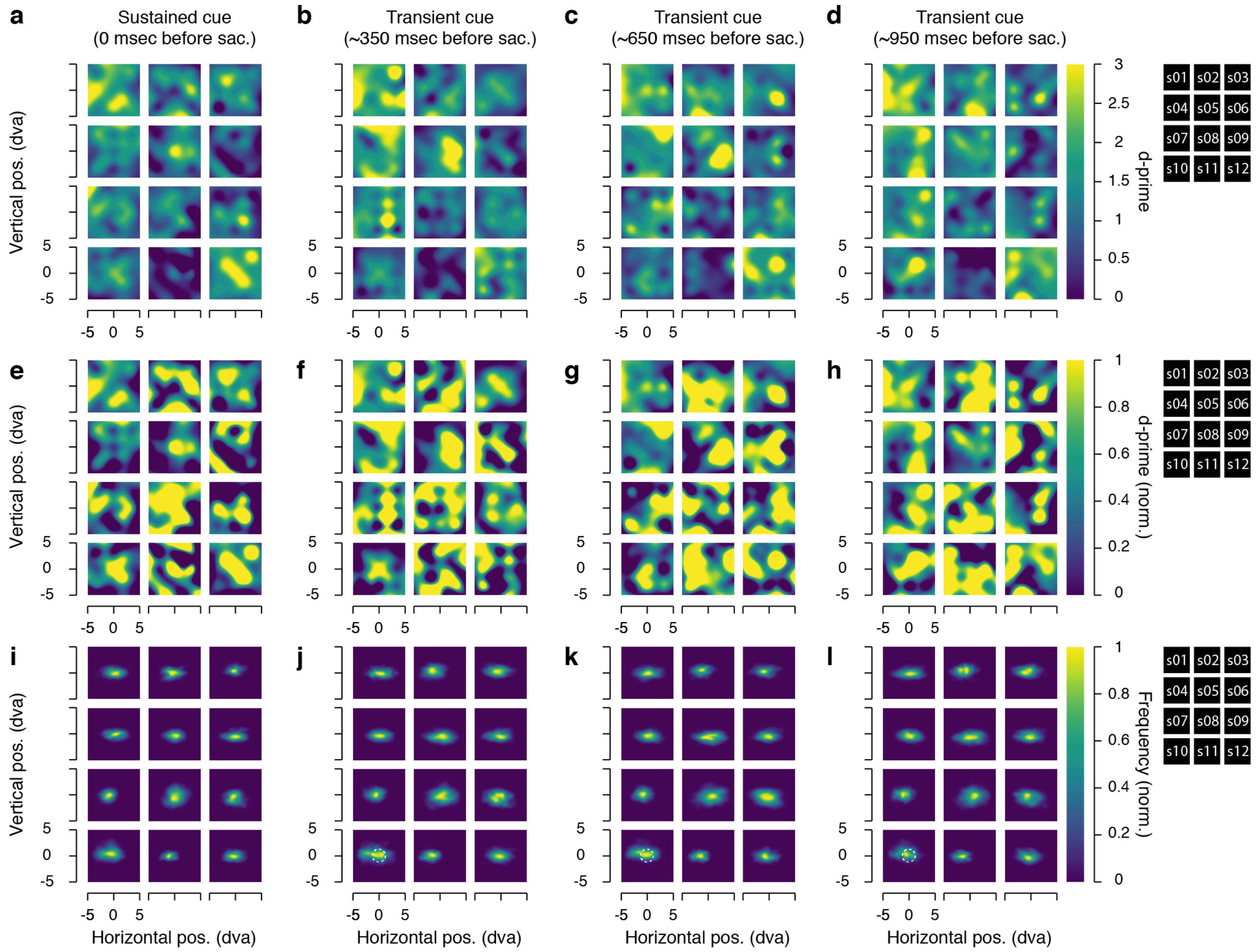

Figure S1. Individual participant presaccadic sensitivity maps and saccade endpoint maps. (**a-d**) Individual participant average sensitivity gathered at 25 positions surrounding the saccade cue (CUE). (**e-h**) Individual participant normalized average sensitivity gathered at 25 positions surrounding the saccade cue (CUE). The normalization allows to highlight difference between conditions rather than individual differences in baseline sensitivity. (**i-l**) Individual participant normalized saccade landing frequency maps. Data are shown for the sustained cue condition (a, e, i) and the transient cue conditions (b-d, f-h, j-l). The transient cue condition is binned in three equal groups of trials where the cue offset preceded the saccade onset by approximately 350 (b, f, j), 650 (c, g, k) or 950 ms (d, h, l). Averaged sensitivity (d'), normalized averaged sensitivity (d' norm.) and normalized saccade landing frequency are shown via color scales. The identity of each participant is indicated with the rightmost inset.

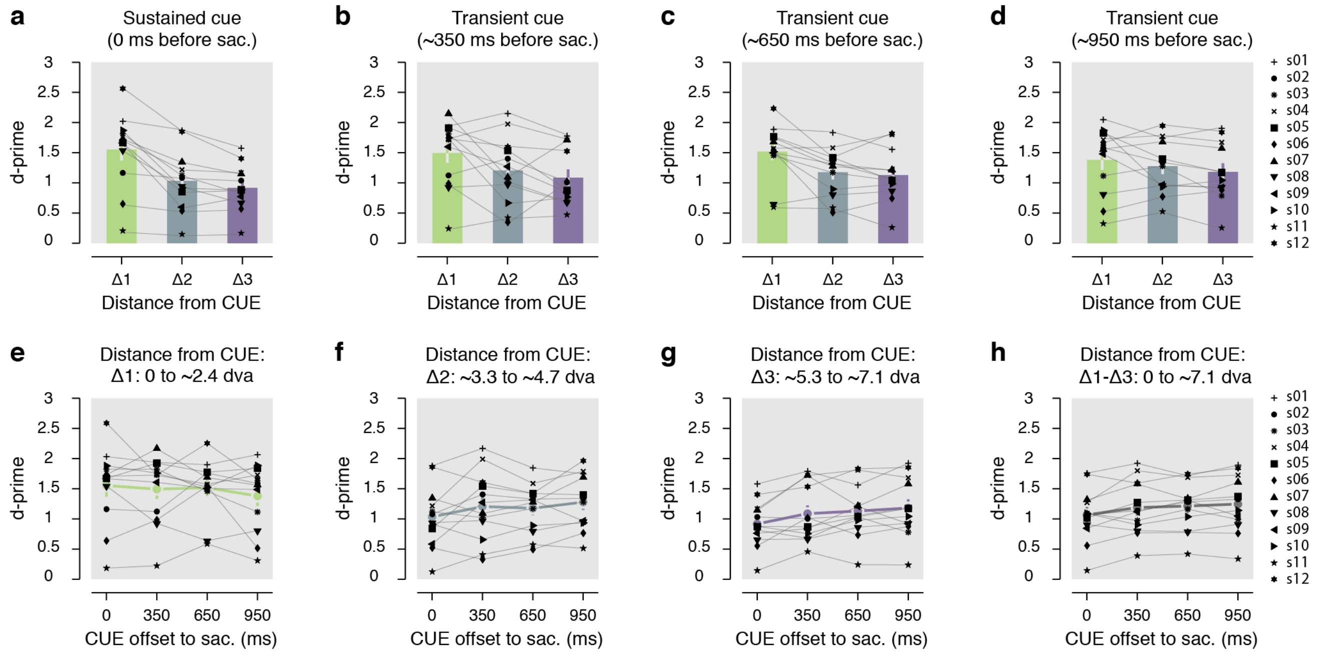

Figure S2. Individual participant results grouped from distance from the cue. **a-d.** Presaccadic sensitivity as a function of the distance from the cue center (Δ1-Δ3). Data are shown for the sustained cue (a) and the transient cue conditions (b-d). The transient cue condition is binned in three equal groups of trials where the cue offset precede the saccade by approximately 350 ms (b), 650 ms (c) or 950 ms (d). **e-h.** Presaccadic sensitivity as a function of the duration between the cue offset and the saccade onset. Data are shown separately for three main distances of the DT from the CUE center (e-g) or for all trials irrespective of their distance from the cue (h). The identity of each participant is indicated by different symbols (see legend). Averaged results are shown in transparent colors, error bars show SEM.

	Sustained cue (0 ms before sac.)				Transient cue (~350 ms before sac.)				Transient cue (~650 ms before sac.)				Transient cue (~950 ms before sac.)			
	Δ1	Δ2	Δ3	Δ1-Δ3	Δ1	Δ2	Δ3	Δ1-Δ3	Δ1	Δ2	Δ3	Δ1-Δ3	Δ1	Δ2	Δ3	Δ1-Δ3
sub-01	2.03	1.88	1.58	1.76	1.93	2.17	1.79	1.92	1.90	1.85	1.56	1.72	2.06	1.75	1.92	1.89
sub-02	1.16	1.08	1.03	1.07	1.12	1.41	1.00	1.14	1.76	1.33	1.06	1.28	1.59	1.34	1.19	1.33
sub-03	1.82	1.12	0.81	1.09	1.82	1.02	0.68	0.97	1.46	1.17	1.03	1.16	1.11	1.28	0.78	0.99
sub-04	1.71	1.21	1.17	1.27	1.73	1.99	1.72	1.80	1.57	1.59	1.81	1.69	1.73	1.79	1.68	1.73
sub-05	1.67	0.84	0.87	1.02	1.92	1.54	0.87	1.27	1.77	1.42	1.04	1.31	1.84	1.40	1.17	1.37
sub-06	0.64	0.52	0.55	0.56	0.98	0.33	1.02	0.77	1.55	0.49	0.73	0.78	0.51	0.76	0.87	0.76
sub-07	1.73	1.34	1.14	1.31	2.16	1.09	1.72	1.58	1.68	1.27	1.21	1.33	1.56	1.68	1.57	1.60
sub-08	1.55	0.94	0.66	0.91	0.93	0.97	0.67	0.80	0.64	0.80	0.86	0.79	0.81	0.96	0.93	0.91
sub-09	1.67	0.58	0.76	0.84	1.61	1.27	0.84	1.12	1.50	1.32	1.21	1.31	1.49	0.97	0.92	1.05
sub-10	1.88	0.91	0.88	1.09	1.76	0.66	0.76	0.90	1.52	0.89	0.98	1.04	1.88	0.93	1.04	1.14
sub-11	0.18	0.12	0.14	0.14	0.22	0.41	0.46	0.39	0.59	0.57	0.24	0.42	0.31	0.51	0.24	0.34
sub-12	2.59	1.86	1.40	1.74	1.73	1.61	1.53	1.59	2.25	1.42	1.83	1.74	1.66	1.96	1.85	1.84

Table S1. Individual participant presaccadic sensitivity results grouped from distance from the cue. Presaccadic sensitivity as a function of the distance from the cue center (Δ1, Δ2, Δ3) or combined across distances (Δ1-Δ3) for the sustained cue (column 1) and the transient cue conditions (columns 2-4). Individual subjects are shown as different lines.

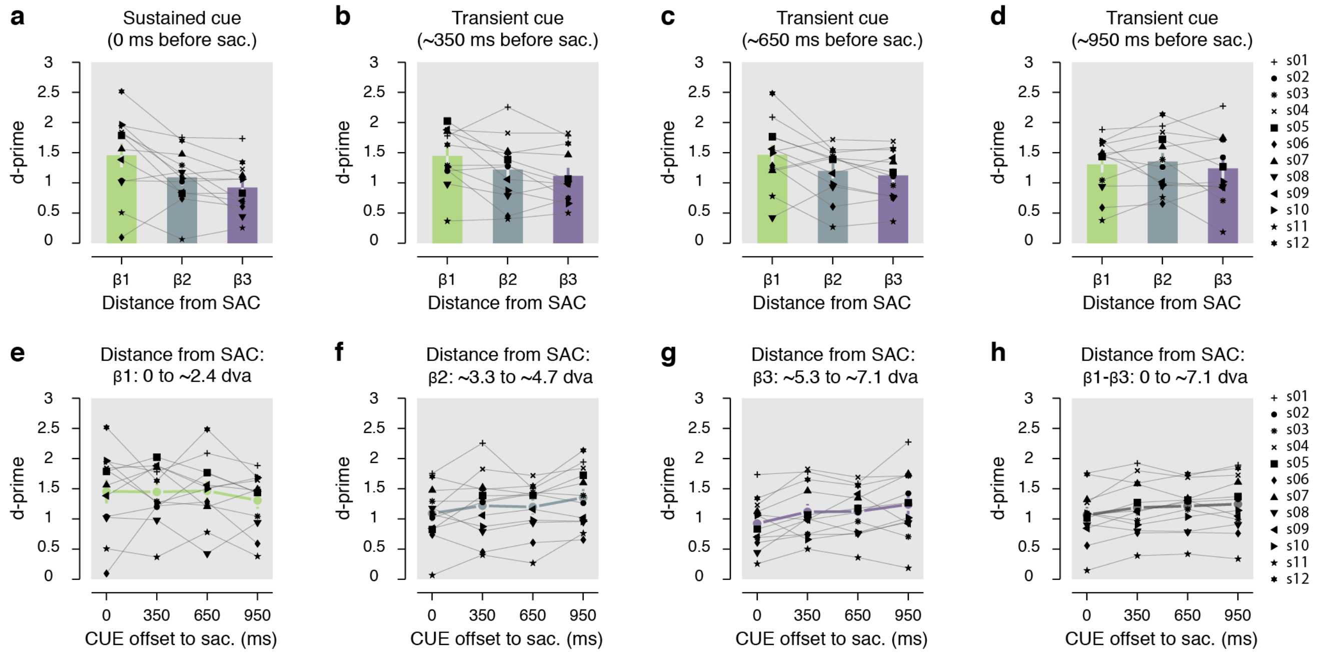

Figure S3. Individual participant results grouped from distance from the saccade endpoint. **a-d.** Presaccadic sensitivity as a function of the distance from the saccade endpoint (β1- β3). Data are shown for the sustained cue (a) and the transient cue conditions (b-d). The transient cue condition is binned in three equal groups of trials where the cue offset precede the saccade by approximately 350 ms (b), 650 ms (c) or 950 ms (d). **e-h.** Presaccadic sensitivity as a function of the duration between the cue offset and the saccade onset. Data are shown separately for three main distances of the DT from the saccade endpoint (e-g) or for all trials irrespective of their distance from the cue (h). The identity of each participant is indicated by different symbols (see legend). Averaged results are shown in transparent colors, error bars show SEM.

	Sustained cue (0 ms before sac.)				Transient cue (~350 ms before sac.)				Transient cue (~650 ms before sac.)				Transient cue (~950 ms before sac.)			
	β1	β2	β3	β1- β3	β1	β2	β3	β1-β3	β1	β2	β3	β1-β3	β1	β2	β3	β1-β3
sub-01	1.95	1.75	1.73	1.76	1.78	2.26	1.78	1.92	2.09	1.5	1.59	1.72	1.88	1.94	2.27	1.89
sub-02	1.04	1.02	1.07	1.07	1.2	1.27	0.98	1.14	1.76	1.41	1.11	1.28	1.43	1.26	1.42	1.33
sub-03	1.81	1.29	0.69	1.09	1.29	1.3	0.75	0.97	1.22	1.44	0.96	1.16	1.04	1.39	0.7	0.99
sub-04	1.84	1.14	1.23	1.27	1.88	1.83	1.83	1.8	1.52	1.72	1.69	1.69	1.64	1.84	1.71	1.73
sub-05	1.79	0.82	0.83	1.02	2.02	1.39	1.07	1.27	1.77	1.39	1.18	1.31	1.43	1.72	1.27	1.37
sub-06	0.1	0.74	0.61	0.56	1.21	0.45	0.74	0.77	1.29	0.61	0.75	0.78	0.59	0.65	0.96	0.76
sub-07	1.56	1.47	1.12	1.31	1.85	1.51	1.46	1.58	1.2	1.41	1.34	1.33	1.48	1.59	1.74	1.6
sub-08	1.02	1.18	0.45	0.91	0.98	0.8	1	0.8	0.43	0.94	0.77	0.79	0.95	0.96	0.94	0.91
sub-09	1.38	0.84	0.7	0.84	1.88	1.06	0.98	1.12	1.56	1.16	1.41	1.31	1.47	1.02	0.92	1.05
sub-10	1.96	1.07	1.06	1.09	1.26	0.87	0.66	0.9	1.5	0.98	0.77	1.04	1.68	0.96	1.02	1.14
sub-11	0.51	0.07	0.26	0.14	0.37	0.4	0.5	0.39	0.78	0.27	0.36	0.42	0.38	0.76	0.18	0.34
sub-12	2.52	1.7	1.34	1.74	1.63	1.49	1.65	1.59	2.48	1.55	1.55	1.74	1.68	2.14	1.72	1.84

Table S2. Individual participant presaccadic sensitivity results grouped from distance from the saccade endpoint. Presaccadic sensitivity as a function of the distance from the cue center (β1, β2, β3) or combined across distances (β1-β3) for the sustained cue (column 1) and the transient cue conditions (columns 2-4). Individual subjects are shown as different lines.

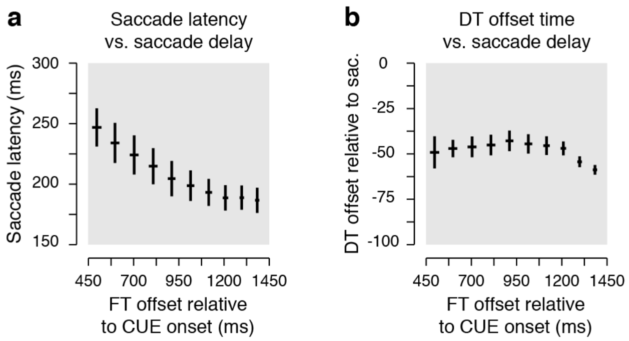

Figure S4. Saccade latency and DT offset time as a function of the saccade signal delay. (**a**) Average saccade latency as a function of the average saccade signal delay (FT offset relative to CUE onset) computed in ten equal trial bins. (**b**) Average DT offset time relative to the saccade onset as a function of the average saccade signal delay computed in ten equal trial bins. By estimating saccade latency as a function of the saccade signal delay at the end of each block, we played the DT on average 48.02 ± 1.16 ms (mean ± SEM) before the saccade. Error bars show SD of their respective axis.
